# The catalytic performance of CuFe_2_O_4_@CQD nanocomposite as a high-perform heterogeneous nanocatalyst in nitroaniline group reduction

**DOI:** 10.1038/s41598-023-28935-z

**Published:** 2023-02-27

**Authors:** Samin Naghash-Hamed, Nasser Arsalani, Seyed Borhan Mousavi

**Affiliations:** 1 Research Laboratory of Polymer, Department of Organic and Biochemistry, Faculty of Chemistry, University of Tabriz, Tabriz, Iran; 2 J. Mike Walker ‘66 Mechanical Engineering Department, Texas A&M University, College Station, TX USA

**Keywords:** Environmental sciences, Catalysis, Inorganic chemistry, Materials chemistry

## Abstract

In this study, we fabricated an economical, non-toxic, and convenient magnetic nanocomposite of CuFe_2_O_4_ nanoparticles (NPs)/carbon quantum dots (CQDs) of citric acid via the co-precipitation method. Afterward, obtained magnetic nanocomposite was used as a nanocatalyst to reduce the ortho-nitroaniline (o-NA) and para-nitroaniline (p-NA) using a reducer agent of sodium borohydride (NaBH_4_). To investigate the functional groups, crystallite, structure, morphology, and nanoparticle size of the prepared nanocomposite, FT-IR, XRD, TEM, BET, and SEM were employed. The catalytic performance of the nanocatalyst was experimentally evaluated based on the ultraviolet–visible absorbance to assess the reduction of o-NA and p-NA. The acquired outcomes illustrated that the prepared heterogeneous catalyst significantly enhanced the reduction of o-NA and p-NA substrates. The analysis of the absorption showed a remarkable decrease for ortho-NA and para-NA at λ_max_ = 415 nm in 27 s and λ_max_ = 380 nm in 8 s, respectively. The constant rate (k_app_) of ortho-NA and para-NA at the stated λ_max_ was 8.39 × 10^–2^ s^−1^ and 5.48 × 10^–1^ s^−1^. The most highlighted result of this work was that the CuFe_2_O_4_@CQD nanocomposite fabricated from citric acid performed better than absolute CuFe_2_O_4_ NPs, since nanocomposite containing CQDs had a more significant impact than copper ferrite NPs.

## Introduction

*Nanocatalysis* is among the ultimate fascinating catalysis category, which has important advantages incorporated with both heterogeneous and homogenous catalysis^1^. The rapid development of chemical industries leads to an increasing number of aromatic pollutants that significantly threaten nature and the environment. Finding an effective way to control aromatic pollutants requires scientific and technological importance to be grown up. Innumerable research studies on the environmental issues of aromatic pollutants have attempted to pay considerable attention to water-soluble aromatic dyes and nitro compounds. Catalytic reduction of aromatic compounds, which are water-soluble by heterogeneous catalysts due to their easy usage, high efficiency, profitability, and low cost, has received much attention. Moreover, catalytic reduction and conversion of nitroaromatic compounds to valuable amino-based compounds have been widely reported. According to the reduction reaction model, such as the reduction of chemical aromatic dyes or nitro compounds by a reductant agent of NaBH_4_, qualitative assessment and comparison of different catalysts based on their activities can also be achieved^2–5^. In recent years, the use of dyestuff outcomes and aromatic pollutants have risen due to industrial modification and technological advancement. Also, discharging the organic pollutants directly into the aqueous environment severely affects aquatic and human life. Many industries, including textile, plastic, cosmetics, and leather, unload their squander into the water resources, which could influence the environment and aquatic life^6^. Nitroaromatic compounds are classified as environmental pollutants since they have been released into the atmosphere^7^. The ortho-nitroaniline (o-NA) and para-nitroaniline (p-NA) have been reported as highly toxic pollutants in wastewater by the United States Environmental Protection Agency (US-EPA). In order to remove or lessen the impact of pollution from water sources, various approaches have been revealed, like adsorption, microbial degradation, photocatalysis, catalysis, electrochemical treatment, and Fenton’s method^8,9^. Among the mentioned methods, catalysis is the greater way to eliminate pollution due to its properties such as time-saving, high satisfaction, and economical.

Recently, nanoparticles have been used in various applications such as heat transfer^10–14^, lubrication^15–17^, CO_2_ capture^18–20^, textile manufacturing^21–25^, photocatalyst, water treatment systems^26^, and catalysis^27^. Magnetic ferrites are used in fundamental research fields due to their electrical, magnetic, chemical/thermal stability properties and spinal properties (MFe_2_O_4_; M = Ni, Co, Fe, Cu, Zn, etc.)^28,29^. The chance of synthesizing the nano ferrites in the form of solid solutions can unclose the proper way to adapt and alter their properties for vast applications^30,31^. Nano-crystalline magnetic ferrite of (CuFe_2_O_4_) has been fabricated by different methods, such as solid-state reaction^32^, co-precipitation^33^, hydrothermal^34^, solution auto combustion^35^, and sol–gel^36^. The intriguing outcomes revealed that the prepared samples’ surface area, magnetic parameter, particle size, crystallites, and catalytic properties were changed based on preparation techniques^37^.

*Carbon* is an adjustable element that could produce various hybridization modes, including sp, sp^2^, and sp^3^. This makes a broad spectrum of allotropes from the softest to the hardest materials^38^. Much theoretical to practical efforts have focused on carbon nanostructures progressing in a broad range in terms of application. Carbon-based dots (CDs) can be classified into CQDs (carbon quantum dots), GQDs (graphene quantum dots), and carbon nanodots (CNDs) based on their carbon arrangement and crystal structure^39,40^. Carbon quantum dots were randomly unearthed throughout the purification stages of carbon nanotubes in 2004^41^. Carbon quantum dots have a broad range of utilization due to their remarkable characteristics, such as low toxicity, higher fluorescence properties, intriguing biocompatibility, greater chemical stability, and electron transfer efficiency^42^.

It is claimed that the reduction product of o-NA and p-NA is o-phenylenediamine (o-PDA) and p-phenylenediamine (o-PDA), respectively, which have been widely used in different fabrication, namely pharmaceuticals, polymers, dyes, surfactants, and pesticides^43^. Edison et al.^44^ studied the ortho-nitroaniline reduction by adding NaBH_4_ and Ag nanoparticles (NPs) fabricated from Tamarindus indica seed. They observed that the green-synthesized Ag NPs had an excellent performance in reducing o-NA with a constant rate of about 2.43 × 10^–3^ s^−1^, which was greater than other synthesized Ag NPs as a candidate catalyst. Baran et al.^45^ examined the o-NA reduction with a practical, highly active, and easily recoverable hybrid Pd/CoFe_2_O_4_/chitosan nanocatalyst. The outcomes represented that the nanocatalyst could convert the o-NA to o-PDA in 65 s, which is an excellent result due to its short reaction time. Gupta et al.^46^ analyzed the impact of gold NPs by synthesizing the shape-selective Au NPs via microwave-assisted technique in 6 min. They noticed that gold nanocubes could reduce the para-NA in 6 min and showed great reusability for at least four cycles. The shape dependency of nanocatalyst was further confirmed in their study. Liu et al.^47^ assessed the persuasive catalyst for reducing p-NA by KBH_4_ with a prepared gold nanoflowers (GNFs) catalyst. The outcomes demonstrated that GNFs catalysts had an efficacious influence on the reduction of p-NA. Different nanocatalysts in shapes, including dog bone, rod, and spherical shapes, were fabricated from gold (Au) by Jiji et al.^48^. They concluded that the gold NPs with a rod shape had a superior effect rather than spherical and dog bone shape NPs. In addition, the reaction time of 42, 122, and 230 s declared for rod, dog bone, and spherical shape, respectively. Revathi et al.^49^ studied the nanocomposite performance by synthesizing the facile and low-cost Cu–CuO nanocomposite to reduce the nitroarenes. They observed that prepared (Cu–CuO) nanocomposite revealed greater catalytic enhancement to reduce the p-NA into the p-PDA rather than Cu and CuO.

According to the conducted literature review, the preponderance of examinations in the aforementioned area announces a positive impact on the catalytic effect of different catalysts with various shapes on the reduction of o-NA and p-NA. Based on the investigations in the field of reduction, comprehensive examinations have been carried out using expensive and rare materials, such as silver and gold. Also, using several materials to prepare nanocomposites is not economically friendly, and it causes much time to fabricate nanocatalysts. Consequently, in this article, for the first time to the best of our knowledge, CuFe_2_O_4_@CQD nanocomposite was successfully synthesized as a catalyst from citric acid and used to reduce organic pollutants of o-nitroaniline and p-nitroaniline.

We planned and fabricated a novel and economical magnetic CuFe_2_O_4_@CQD nanocomposite as a nanocatalyst via the co-precipitation method. The impact of the nanocatalyst on the reduction of o-NA and p-NA with a reductant of NaBH_4_ was analyzed. To evaluate the reusability of the nanocatalysts, magnetic catalyst NPs were separated from the solution by an external and robust magnet and were reused for up to at least 6 recycles.

## Experimental part

### Chemicals

Citric acid (HOC(CO_2_H) (CH_2_CO_2_H)_2_), sodium borohydride (NaBH_4_), para-nitroaniline (C_6_H_6_N_2_O_2_), ethanol (C_2_H_5_OH), sodium hydroxide (NaOH), and ortho-nitroaniline (C_6_H_6_N_2_O_2_) were commercially purchased from *Merck*.

Scrutinizing the functional groups of fabricated CQDs and nanocomposite of CuFe_2_O_4_@CQD were employed by Fourier transform infrared spectroscopy [FT-IR, Nexus Model Infrared spectrophotometer (Nicolet Co, USA)]. A SEM (Carl Zeiss 1430VP L, Germany) and TEM (EM 208S, Philips, Netherland) were applied to illustrate nanocomposite morphology and size distribution. To obtain the crystal structure and crystallite size of nanoparticles at room temperature, X-ray diffraction (XRD, PW1730, Philips, The Netherlands) with Cu-Kα (λ = 1.54056 A°) radiation was performed. The pattern was analyzed with XRD, recorded with a step size of 0.05°/1 s, and between the angular radiuses of 10–80° (2θ). To demonstrate the catalytic property of nanocomposite, UV–visible absorption spectra (UV–Vis, Specord 250, Analytik Jena) was utilized. The magnetic stirrer (Fan Azma Gostar, Iran) was utilized for dissolving the solutions to intercept the agglomeration. A centrifuge (Hettich Centrifuge EBA III) was exploited to help the separation process. Physical adsorption and desorption of N_2_ at 77 K with BELSORP MINI II device was used to measure the BET surface area and pore volume distribution of BJH of the synthesized sample. To evaluate the magnetic properties of the synthesized samples in a magnetic field of ± 15 kOe, a vibrating sample magnetometer (VSM-model MDKF-FORC/VSM, Megnatis-Daghigh-Kashan Co., Kashan, Iran) was utilized. The magnetization as a function of temperature (M–T curves) was recorded in the temperature range of 10–380 K.

### Synthesis of copper ferrite nanoparticles

CuFe_2_O_4_ NPs were synthesized via the facile one-step hydrothermal method. Initially, 0.574 g of tetrasodium EDTA salt as a chelator was dispersed in 50 mL of deionized (DI) water for 10–15 min in an ultrasonic bath cleaner. Then, the stoichiometric amount of 3.24 g of anhydrous FeCl_3_ and 1.35 g of CuCl_2_·2H_2_O were added to the desired solution under constant stirring speed. The alkaline solution, prepared by adding 17.7 g of granular sodium hydroxide per 100 mL of water, was added dropwise to the solution using a syringe (during the reaction, the pH of the reaction was continuously monitored using a pH meter). When the pH reached 10–11, the solution was poured into a Teflon autoclave at 185 °C for 15 h. After the completion of the reaction by a strong external magnet, the magnetic sediment was separated and washed several times with ethanol and distilled water. Finally, the prepared brown sediment was dried at 60 °C for approximately 3–4 h.

### Facile fabrication of carbon quantum dots (CQDs) from citric acid

**Figure 1:**
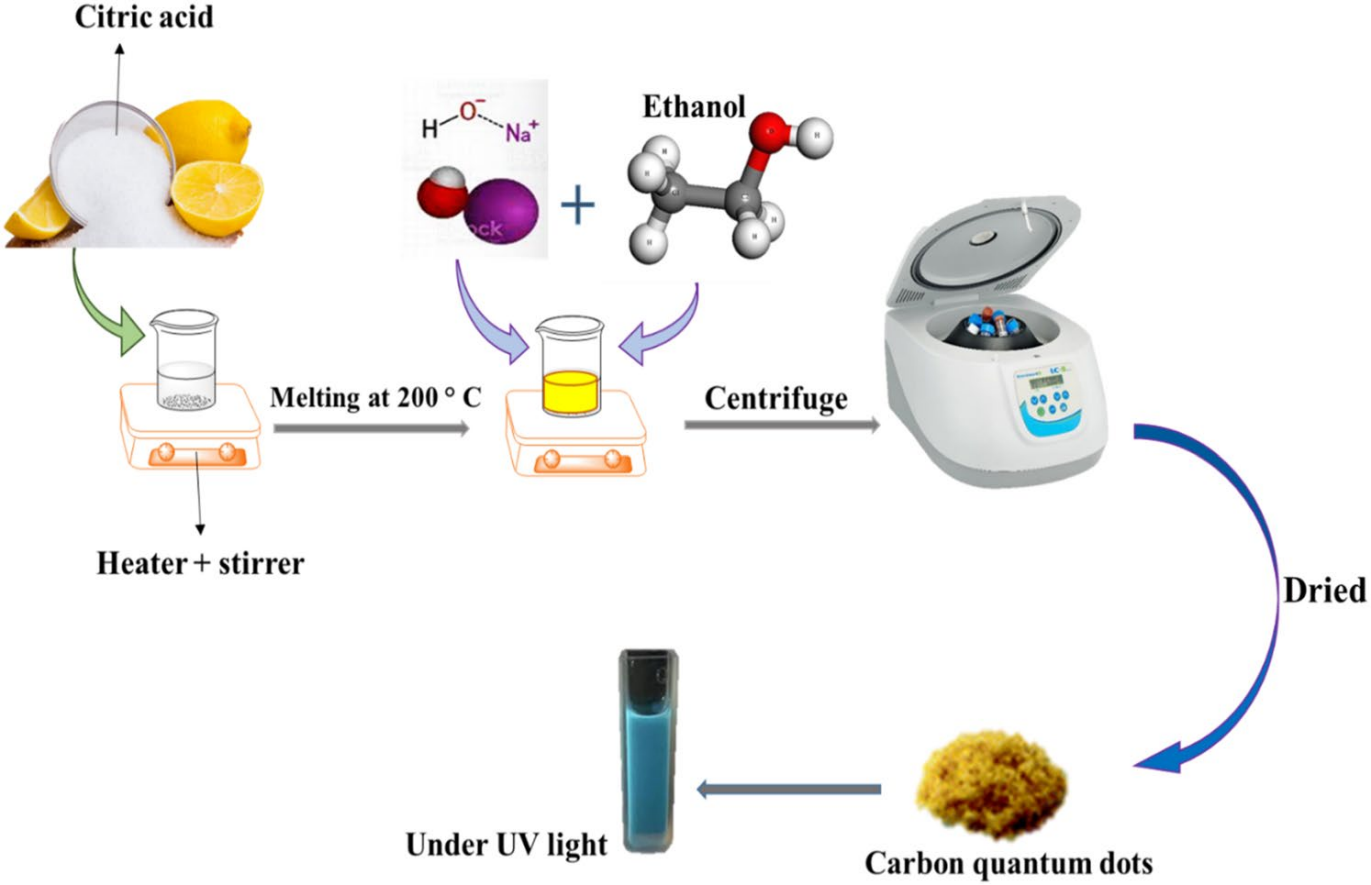
Synthesis of yellowish carbon quantum dots from citric acid.

Fabricating yellowish carbon quantum dots from citric acid is shown in Fig. 1. To prepare quantum dots of carbon, about 2.00 g of citric acid (CA) was melted at 200 °C under continuous magnetic stirring. The prepared NaOH solution (1.00 g of NaOH added to the 100 cc of absolute ethanol) was steadily poured into the CA. When the precipitate formed, the resulting liquid was centrifuged at 7 rpm. Ultimately, the precipitate was dried in an oven at 50 °C for 4 h till the yellowish powder was obtained. Acquired CQDs under UV light had a fluorescence property.

### Fabrication of CuFe_2_O_4_@CQD nanocomposite

**Figure 2:**
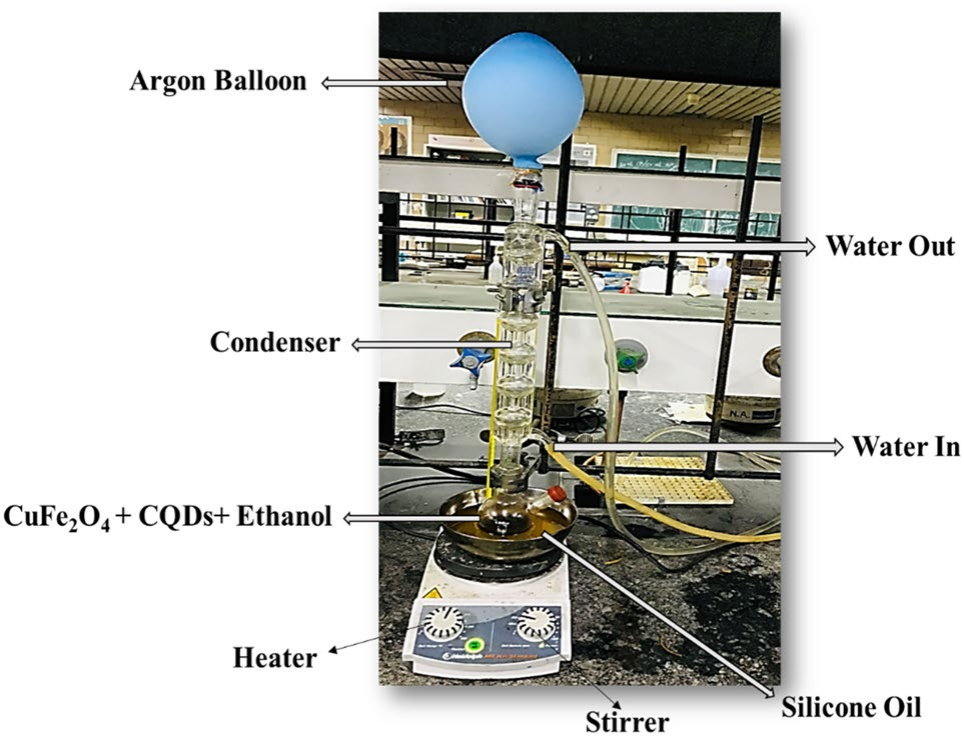
Representation of co-precipitation technique applied for fabrication of CuFe_2_O_4_@CQD nanocomposite.

CuFe_2_O_4_@CQD nanocomposite was fabricated via a simple co-precipitation method with a weight ratio of 2:1. Figure 2 illustrates the schematic of the preparation procedure. In order to fabricate the nanocomposite, 0.105 g of CuFe_2_O_4_ and 0.058 g of CQDs were poured into the 15 mL of ethanol; then, they were dissolved for 15 min. Subsequently, the mixture was heated at 70 °C, incorporated under the argon noble gas, and stirred perfectly under stirring for 5 h into an oil bath. The brown resulting precipitate was detached via an external magnet and alternately washed with deionized (DI) water and ethanol. Finally, it was dried in the hot oven for 4 h at 80 °C. Consequently, CuFe_2_O_4_@CQD nanocomposite was fabricated and ready to use as a reduction reaction catalyst.

### Catalytic performance evaluation

The catalytic reduction examination was employed in the omission and presence of catalyst into a quartz cuvette with a total volume of 3.50 mL and 1 cm path length. This evaluation was conducted to reduce o-nitroaniline and p-nitroaniline as a model of the organic nitro compounds in the presence of NaBH_4_. For a typical investigation, 20 mg of o-NA and p-N were separately dissolved in 100 mL of DI water for 20 min. Afterward, 5 mL of the prepared nitro mixture, 100 mg of NaBH_4_ as a reducing agent, and 3.5 mg of nanocomposite as a catalyst were subsequently added to the testing tube. In the end, 100 μL of the resulting solution was transferred expeditiously into the quartz cuvette, and its absorption was measured once a few seconds till the color of the solution was completely altered.

## Results and discussion

### Characterization

**Figure 3:**
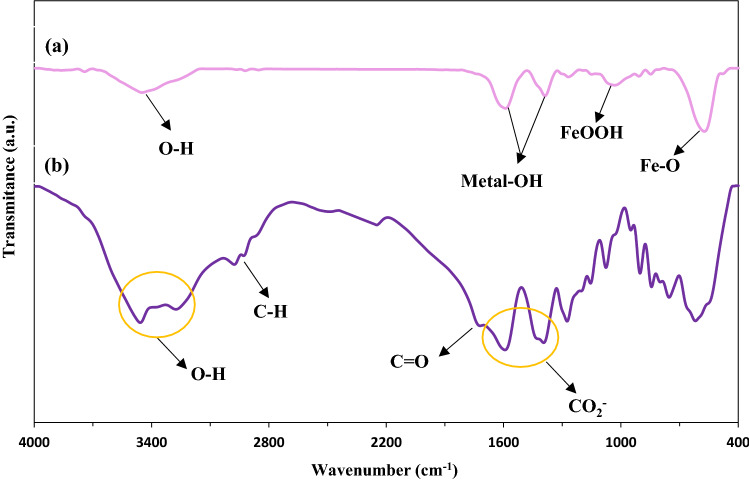
FT-IR spectrum of (**a**) CuFe_2_O_4_@CQD, (**b**) CQDs of citric acid.

Figure 3a shows the FT-IR analysis of the CuFe_2_O_4_@CQD nanocomposite. It is observable in Fig. 3a, that the strong band at approximately 572 cm^−1^ was ascribed to the deformation of Fe–O in octahedral sites, displaying the functional groups of CuFe_2_O_4_ into the CuFe_2_O_4_@CQD nanocomposite^50^. The observed bands at 1388 cm^−1^ and 1595 cm^−1^ corresponded to the metal-OH (Fe-OH and Cu-OH) bending vibration, respectively^51,52^. The intense peak at 1014 cm^−1^ depicted the FeOOH bond. The broad absorption from 3700 to 3400 cm^−1^ corresponded to the O–H stretching vibration group. This stretching vibration belonged to the CuFe_2_O_4_@CQD and was assigned to the occupancy of water molecules that could exist on their surfaces^53^. It is noteworthy that the presence of CQDs in the structure of the considered nanocomposite caused an overlap that enhanced the peak intensity and decreased it compared to the CuFe_2_O_4_ NPs peaks reported in previous work^54^. Meanwhile, some of the peaks disappeared in the FT-IR of the nanocomposite.

The seen IR band in 3400 cm^−1^ in Fig. 3b was ascribed to the CQDs of citric acid, which was attributed to the attendance of the hydroxyl (R-OH) group in the structure of citric acid^55^. As can be seen, the peak at 1728 cm^−1^ was allocated to the carbonyl groups (C=O bond) on the carbon quantum dots^56^. The vibrational band of CH_2_ and the stretching peak of C–OH are seen in 621 cm^−1^ and 1082 cm^−1^, respectively^57^. The intense peaks at 1385 cm^−1^ and 1585 cm^−1^ depicted the symmetric and anti-symmetric vibration of the (CO_2_^−^) group, sequentially, which was consistent with the presence of the acidic group on the surface of the quantum dots of carbon^58,59^. The peak at 2970 cm^−1^ corresponded to the C–H band^60^.

The X-ray diffraction pattern of CuFe_2_O_4_@CQD nanocomposite is represented in The peak demonstrated around 2θ = 24° was not recognized in the X-ray diffraction pattern of CuFe2O4 NPs, which was demonstrated as the CQDs peak.

**Figure 4:**
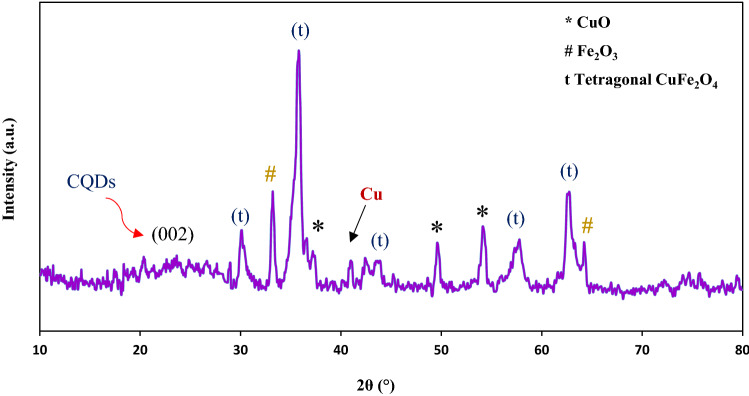
X-ray diffraction pattern of CuFe_2_O_4_@CQD nanocomposite.

Figure 4 can be seen that the XRD pattern for the sample has prime 5 intense peaks at 2θ = 30.12°, 35.84°, 43.99°, 57.74°, and 62.79° corresponded to (112), (211), (312), (112), and (400), consecutively^61^. Every single diffraction of the fabricated sample is in high approval of the JCPDS card (34-0425), illustrating the tetragonal structure of copper ferrite NPs. Furthermore, the peaks seen at 2θ = 37.39°, 49.59°, and 54.14° corresponded to the (*111), (*−202), and (*020), indicating the monoclinic CuO phase plane (JCPDS No. 80-1916)^62^. In terms of Fe^3+^ and Cu^2+^ complex constants, the presence of CuO may be reasonable. The complex constant of Fe^3+^ (log β = 20.19 ± 0.02) is larger than that of Cu^2+^ (log β = 8.08), so Fe^3+^ complexes should be more stable than Cu^2+^. Therefore, Cu^2+^ agglomerates with NaOH and finally forms CuO^53,63^. Some secondary impurities, such as Fe_2_O_3_ were still found, peaks seen at 2θ = 33.69° and 64.24°, which may be attributed to the insolubility of FeO, are in a good acceptance of the JCPDS card (33-0664)^63^. The strong peak at 2θ = 40.97° is indexed to the Cu element^64^.

To enumerate the crystallite size of CuFe_2_O_4_@CQD nanocomposite, the Debye–Scherrer’s formula could be calculated as follows^65,66^:

1 $$D= \frac{K\uplambda }{\beta \mathrm{cos}\theta}$$

where ${D}_{avg}$, $K$, $\uplambda$, $\beta$, and $\theta$ are ascribed to the average crystallite size of NPs (nm), the wavelength of the X-ray pattern, the full width at the half apex of the maximum diffraction (FWHM), and the Bragg angle. This peak corresponded to the reflection of the (002) plane of carbon quantum dots, confirming the accurate fabrication of nanocomposite. Moreover, it is obvious that the sharp peaks denoted the crystallite and purity nature of fabricated samples, and the broad peak of CQD represented the amorph structure^67^. According to the FWHM crystal plane (211), based on the Scherrer equation, the crystallite size of nanoparticles was calculated to be about 14.30 nm. The peak demonstrated around 2θ = 24° was not recognized in the X-ray diffraction pattern of CuFe_2_O_4_ NPs, which was demonstrated as the CQDs peak.

**Figure 5:**
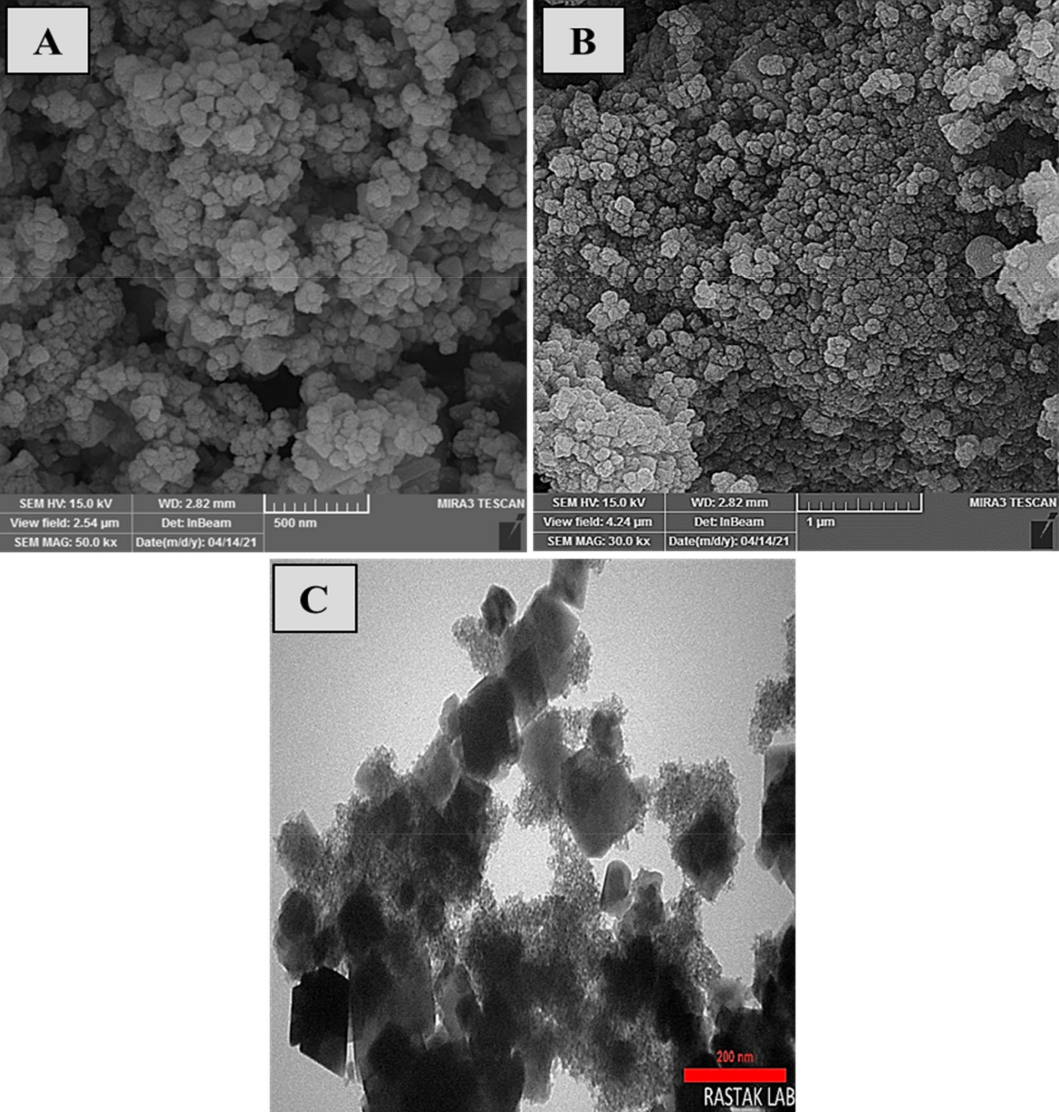
(**A**, **B**) SEM, (**C**) TEM images of CuFe_2_O_4_@CQD nanocomposite.

**Figure 6:**
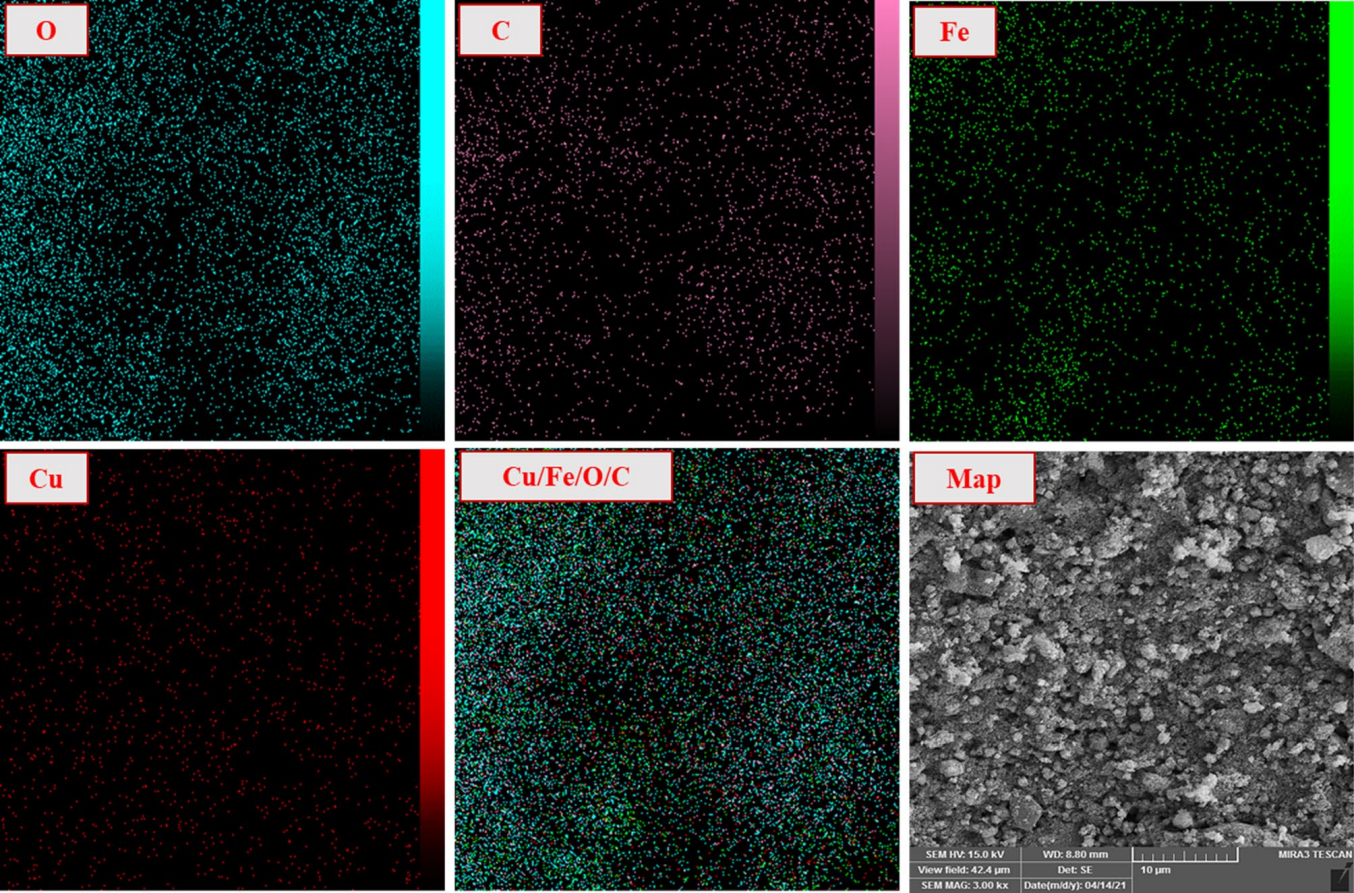
EDS elemental mapping of CuFe_2_O_4_@CQD nanocomposite.

**Figure 7:**
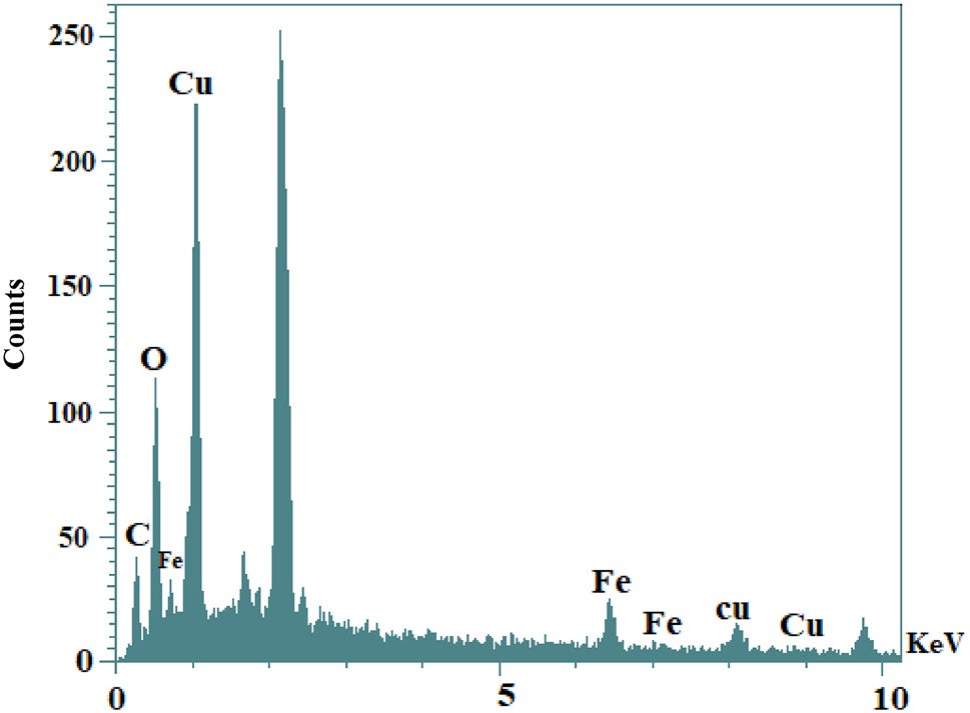
The EDX spectrum of CuFe_2_O_4_@CQD nanocomposite.

The surface structure, morphology, shape, and crystallite of CuFe_2_O_4_@CQD nanocomposite were analyzed using SEM and TEM (Fig. 5). Moreover, acquired nanocomposite depicted a mixture of cubic and spherical structures due to synthesizing techniques^66^. The CuFe_2_O_4_@CQD nanocomposite had a diameter range of 78.5 nm. It becomes apparent that pictures depicted agglomeration due to the two dominant reasons. The CuFe_2_O_4_ NPs have a magnetic property, and carbon quantum dots tend to build new larger structures, so they stay in one place to get bigger and are seen as agglomeration. The outcome of this occurrence is that the smallest particles accumulate at higher temperatures and form large particles that are heterogeneous in size^67^. The EDS-mapping pictures of the nanocatalyst are illustrated in Fig. 6, which confirms the elemental composition of CuFe_2_O_4_@CQD. Cu, O, C, and Fe elements were well dispersed into the CuFe_2_O_4_@CQD nanocomposite. The EDX spectrum of the CuFe_2_O_4_@CQD nanocomposite is exhibited in Fig. 7. In Fig. 7, the presence of C (citric acid), Cu, Fe, and O elements in the nanocomposite structure is confirmed.

**Figure 8:**
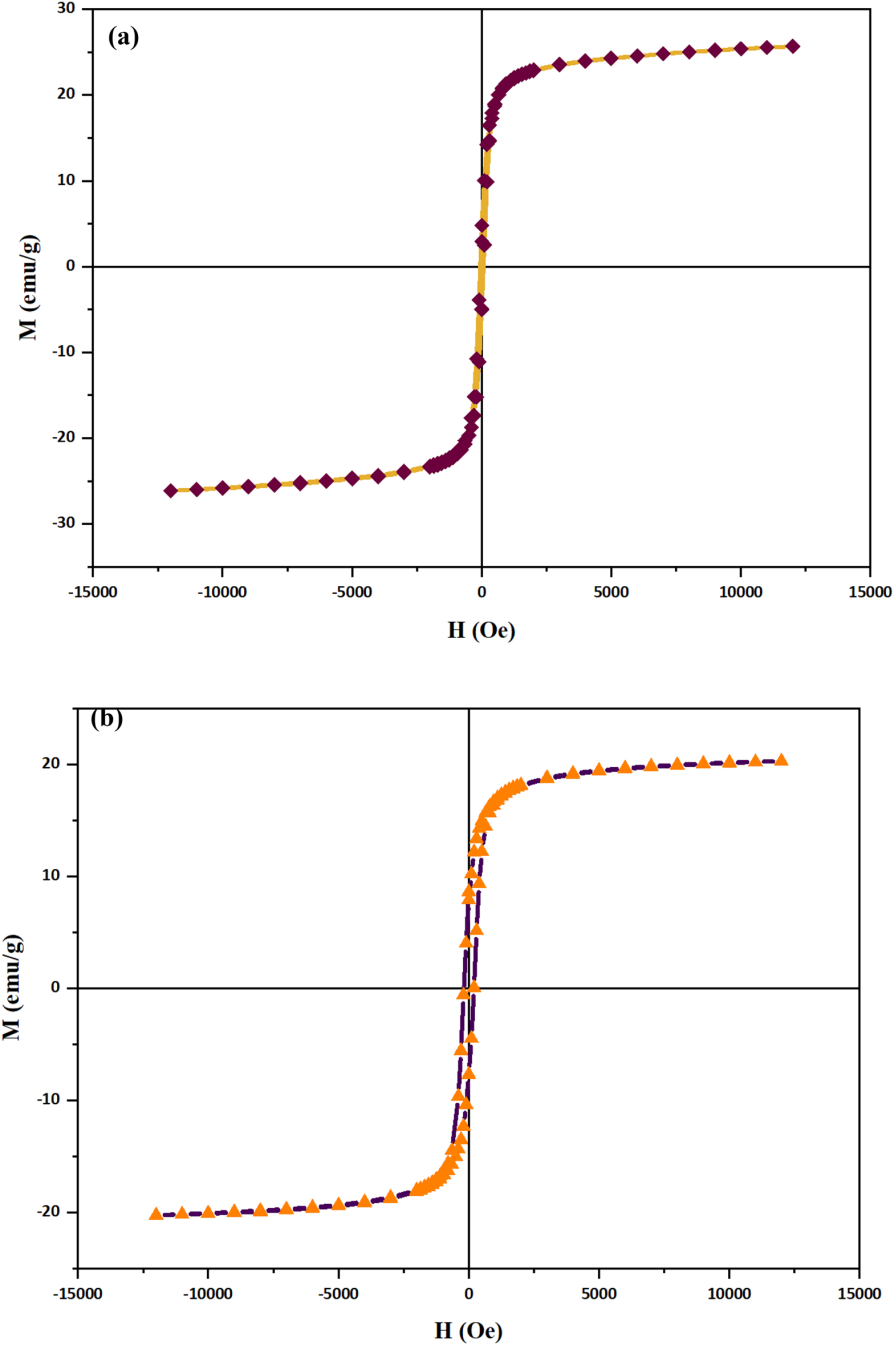
VSM curve of (**a**) CuFe_2_O_4_ nanoparticles, (**b**) CuFe_2_O_4_@CQD nanocomposite.

Corresponding magnetic measurements of CuFe_2_O_4_@CQD nanocomposite, which were performed in the magnetic field in the range of − 15 to + 15 kOe (M–H hysteresis loop), proved their satisfactory saturation magnetization (M_s_) and favorable superparamagnetic properties (Fig. 8). The M_s_ value of CuFe_2_O_4_ NPs and CuFe_2_O_4_@CQD nanocomposite was 25.68 emu g^−1^ and 20.27 emu g^−1^ without observing apparent remanence and coercivity, respectively. The CuFe_2_O_4_ NPs have a higher saturation magnetization than CuFe_2_O_4_@CQD nanocomposite. Such NPs can be rapidly assembled by an external magnetic field, providing multifunctional properties for separation and reduction processes. Because the average particle size of samples was less than 20 nm and showed reversible hysteresis curves with negligible retention and coercivity, the sample could exhibit superparamagnetic behavior. The H_c_ for CuFe_2_O_4_ NPs and CuFe_2_O_4_@CQD nanocomposite were 0.064 Oe and 0.7 Oe, sequentially. It has been reported that the lower magnetization in nanoparticles is due to surface spin caused by competitive antiferromagnetic interactions^68^. Lower magnetization can also be caused by the unsaturation of small arbitrarily dispersed particles that exhibit high crystal magnetic anisotropy. Other reasons were considered to justify the lower magnetization in nanoparticles, such as creating a passive magnetic layer, spin glass properties, and irregular cation distribution on the surface of nanoparticles^69^.

Table 1 demonstrates the BET/BJH parameters of CuFe_2_O_4_@CQD nanocomposite and CuFe_2_O_4_ nanoparticles. Based on the acquired features, CuFe_2_O_4_@CQD nanocomposite has 29.5 m^2^/g of surface area, 0.04 cm^3^/g of pore volume, and 5.67 nm of mean pore diameter, revealing its nanoporous associated with significant accessible surface area. The free surface area of 110.6 m^2^/g and pore volume of 0.25 cm^3^/g disclose the considerable porosity of the synthesized CuFe_2_O_4_ nanoparticles. Additionally, the mean pore volume of 7.3 nm indicated its nanostructure, as expected.

**Table 1:** Structural and textural properties of the prepared materials.

Material	BET surface area (m^2^/g)	BJH pore volume (cm^3^/g)	Mean pore dimeter (nm)
CuFe_2_O_4_@CQD nanocomposite	29.5	0.04	5.67
CuFe_2_O_4_ nanoparticles	110.6	0.25	7.3

**Figure 9:**
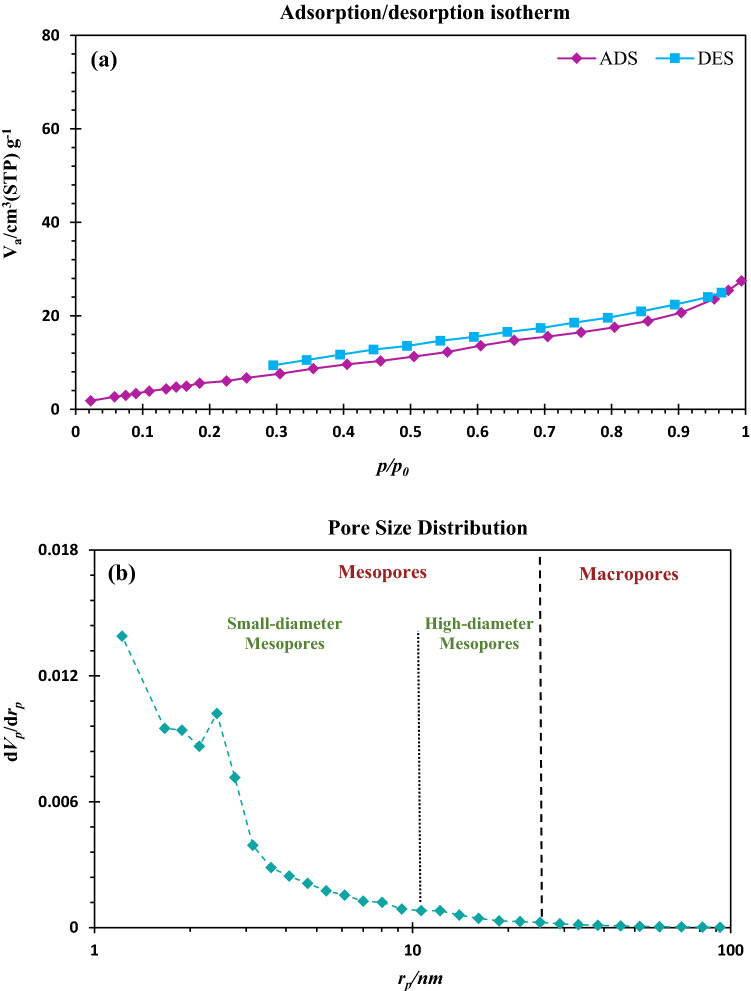
(**a**) N_2_ adsorption/desorption isotherm, (**b**) pore size distribution curve of CuFe_2_O_4_/CQD nanocomposite.

N_2_ adsorption/desorption isotherm of CuFe_2_O_4_@CQD nanocomposite in P/P_0_ ranging from 0 to 1 is presented in Fig. 9a. CuFe_2_O_4_@CQD nanocomposite indicates the trend classified as II mode, indicative of nanoporous structure. The hysteresis of H4 is obvious for CuFe_2_O_4_@CQD nanocomposite, indicating slim slit-like pores and wide distribution of irregular internal mesopores in particles. This mode and hysteresis simultaneously reveal the formation of mesopores in the nanoporous CuFe_2_O_4_@CQD nanocomposite. Figure 9b illustrates the BJH pore size distribution of CuFe_2_O_4_@CQD nanocomposite at pore diameters ranging from 1 to 100. This curve indicates the wide presence of small-diameter mesopores, minor high-diameter mesopores, and the paucity of macropores in CuFe_2_O_4_@CQD nanocomposite texture. There is substantial conformity between pore size distribution and N_2_ adsorption/desorption of CuFe_2_O_4_@CQD nanocomposite, which possesses the nanoporous structure to teem with small-diameter mesopores.

**Figure 10:**
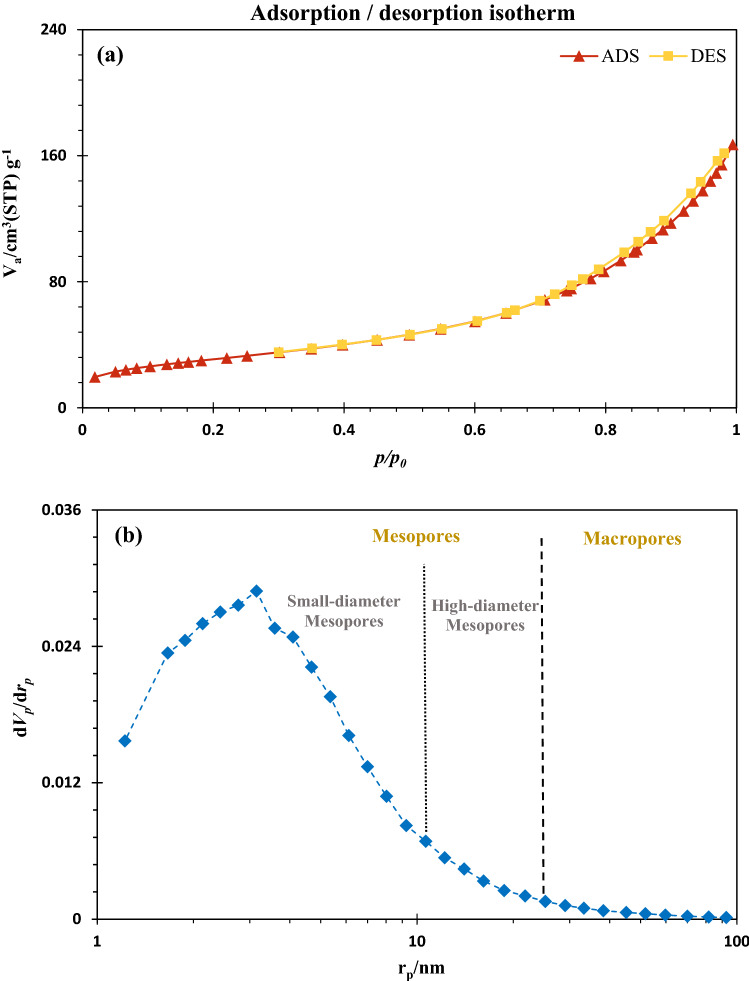
(**a**) N_2_ adsorption/desorption isotherm, (**b**) pore size distribution curve of CuFe_2_O_4_ nanoparticles.

The N_2_ adsorption/desorption and pore size distribution profile of CuFe_2_O_4_ NPs are depicted in Fig. 10. Illustrated N_2_ adsorption/desorption isotherm of CuFe_2_O_4_ NPs in P/P_0_ ranging from 0 to 1 demonstrates the II mode trend, indicating the formation of nanoporous configuration. Furthermore, the hysteresis of this nanoparticle can be classified as H4, which corroborates the slim slit-like pores and the scattering of inner mesopores in texture. Consequently, based on the reported BJH pore volume profile and N_2_ adsorption/desorption plot, it can be concluded that CuFe_2_O_4_ NPs constitute the mesopores and identifies as the mesoporous configuration. The presented pore volume profile indicates the presence of mesopores, with a pore diameter of less than 50 nm, in the texture of the nanoparticles. Moreover, there is no formation of macropores during the development of corresponding nanoparticles. As a result, this nanoparticle is a mesoporous structure.

### Catalytic examinations

**Figure 11:**
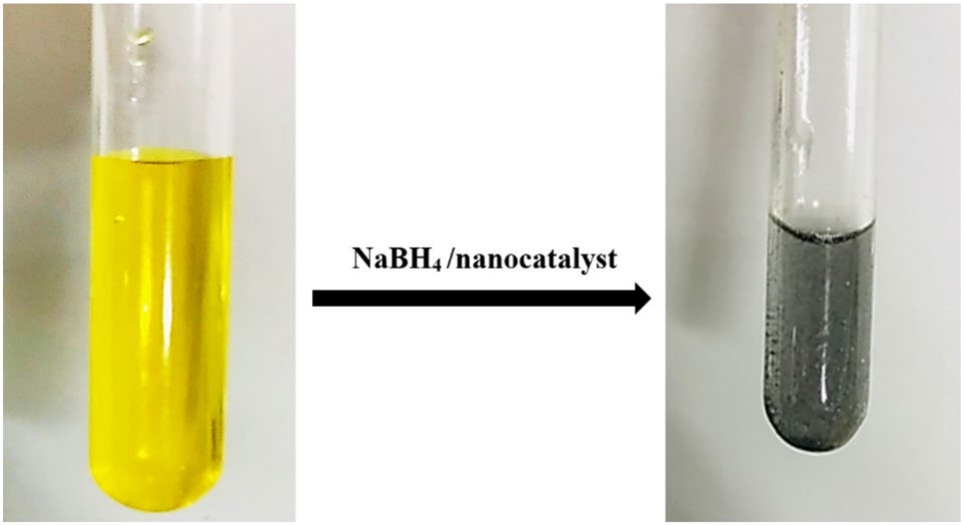
The colour transformation of ortho-nitroaniline after adding the nanocatalyst.

The catalytic evaluation was measured by UV–Vis spectroscopy at a wavelength between 250 and 550 nm. To inspect the influence of the obtained nanocatalyst on the reduction of o-NA and p-NA as a sample of regenerative organic matter in the water-based solution, a constant amount of acquired catalyst was added after the addition of NaBH_4_ as a reducing agent. Subsequently, to acquire the reduction progress, the decrease in absorbance was calculated as a function of the expressed wavelength^70^. In addition, according to Fig. 11, by monitoring the color change of o-nitroaniline from orange/yellow to light gray (colorless) during the catalytic reduction, we can easily observe that the o-nitroaniline reduction reaction was successfully done. Studies revealed that the main color of o-nitroaniline was dark yellow; after adding NaBH_4_ and nanocatalyst, its color was converted into a colorless solution^45^.

**Figure 12:**
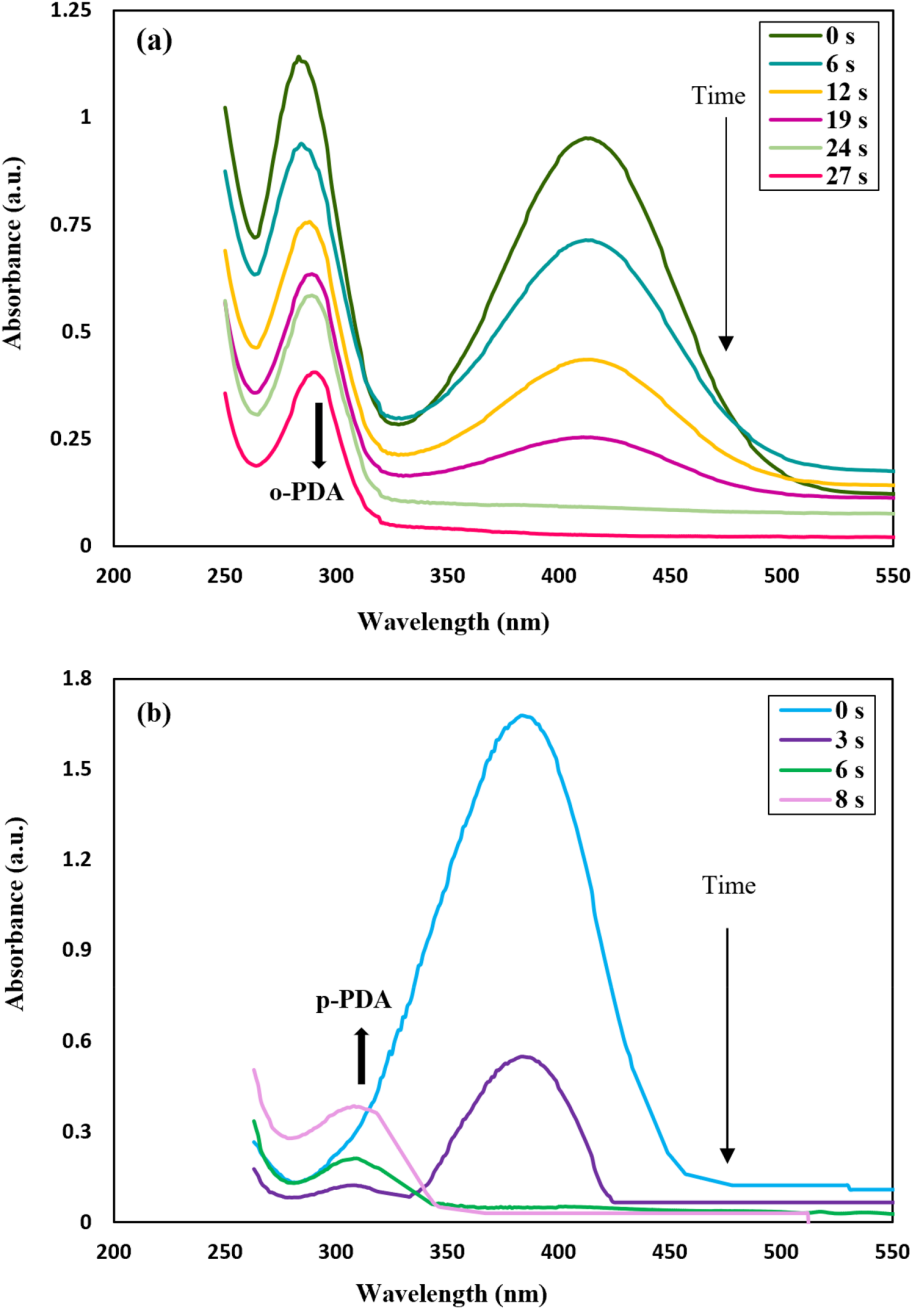
UV–Vis adsorption of (**a**) o-NA to o-PDA, (**b**) p-NA to p-PDA; after adding reductant of sodium borohydride and CuFe_2_O_4_@CQD nanocomposite as a heterogeneous catalyst.

In Fig. 12a, following the addition of the nanocatalyst, adsorption was dropped from about 0.947 to 0.025 at approximately λ_max_ = 415 nm over 27 s because of the diminishing concentration of o-NA. Besides, the elucidated shift from 285 nm to approximately 290 nm can be seen due to the formation of o-PDA^71^. Figure 12b shows two prominent peaks at 285 and 415 nm ascribed to the o-nitroaniline^72^. After adding the fabricated nanocatalyst, by observing a change in the adsorption of p-NA, led to the discovery of an effective catalyst that catalyzed p-nitroaniline. Moreover, when CuFe_2_O_4_@CQD nanocomposite was added to the aqueous solution as a catalyst, the adsorption declined from 1.67 to 0.023 at 380 nm within 8 s. The peak, which increased from 0.193 to 0.380 at 307 nm, was attributed to the new product, para-PDA.

**Figure 13:**
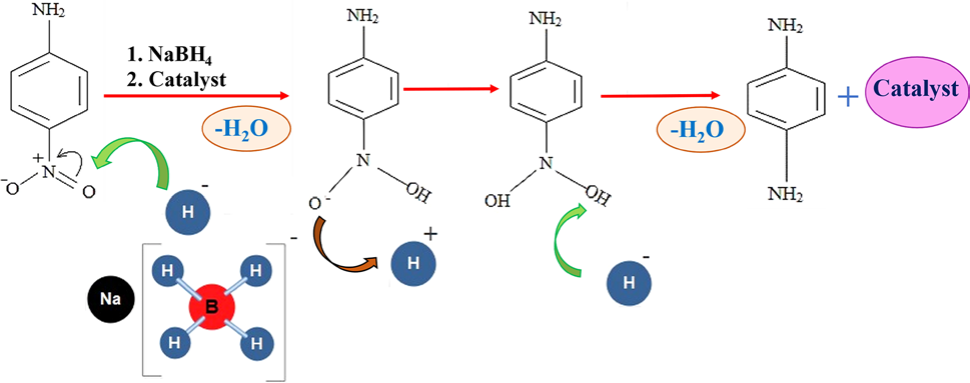
Mechanism of para-nitroaniline catalysed by the prepared nanocatalyst.

Considering Fig. 13, at the starting point, o-NA, along with NaBH_4_ as an electron donor (reducer), was adsorbed on the outside of the fabricated nanocatalyst, so metal nanoparticles were charged and intensified the hydrogenation of o-NA. Thus, the nitroarenes were converted to a nitroso compound and reduced to a hydroxylamine compound. Ultimately, the hydroxylamine composition changed swiftly to the aromatic amine composition^73^.

One commonly utilized equation for calculating the conversion rate of nanocatalyst is to reduce considered o-nitroaniline to o-phenylenediamine and p-nitroaniline to p-phenylenediamine. The conversion ratio of the reduction reaction of o-NA and p-NA was calculated using Eq. (2)^73^.

2 $$\mathrm{Conversion }(\%)=\frac{{A}_{0}-{A}_{t}}{{A}_{0}}\times 100$$

where ${A}_{0}$ is the absorbance of o-nitroaniline and p-nitroaniline at the starting time and ${A}_{t}$ is the absorbance of nitroaromatics in any time of t.

### Evaluate the reduction rate of o-nitroaniline and p-nitroaniline

**Figure 14:**
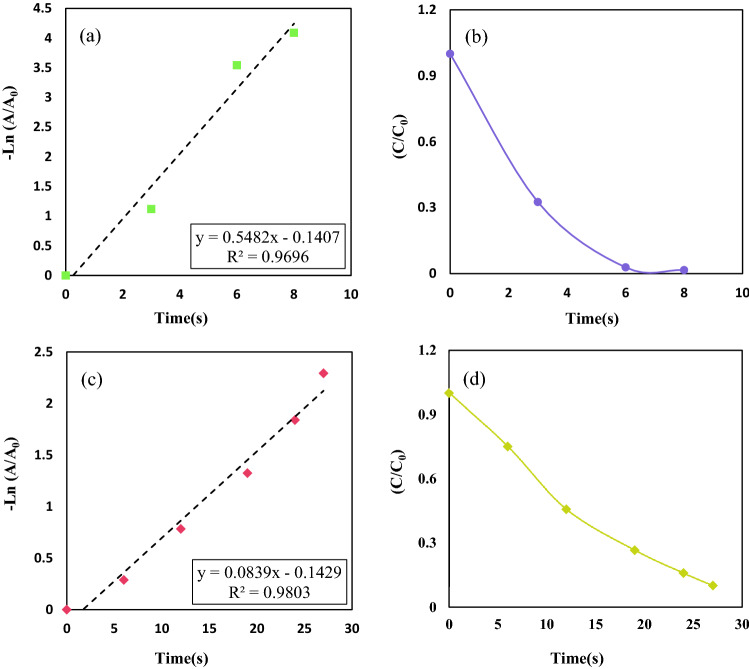
Plots of $-\mathrm{ln}(\frac{{A}_{t}}{{A}_{0}})$ vs time for the reduction reaction of (**a**,**b**) o-nitroaniline to ortho- PDA, (**c**,**d**) p-nitroaniline to para- PDA using prepared nanocatalyst.

The concentration of NaBH_4_ as a reducing agent is quite higher than the nanocatalyst amount; therefore, the reaction rate relied upon the concentration of o-NA and p-NA. Consequently, the reduction illustrated the first-order kinetics. We utilized Eq. (3) to evaluate the catalytic reduction reaction of ortho-nitroaniline along with para-nitroaniline^9^:

3 $$\mathrm{ln}\left(\frac{{C}_{t}}{{C}_{0}}\right)=\mathrm{ln}(\frac{{A}_{t}}{{A}_{0}})=-{K}_{app}\cdot t$$

where ${C}_{t}$ or ${A}_{t}$ is the concentration/absorbance measured at time t in the stated wavelength, ${C}_{0}$ or ${A}_{0}$ denote the concentration/absorbance of nitroaromatic at the first time when the reaction was not completely started, and $t$ shows reaction time at any time. ${K}_{app}$ is the constant rate of nanocatalyst. All the detailed information about the reduction of o-nitroaniline and p-nitroaniline catalyzed by nanocatalyst was calculated and represented in Fig. 14 and Table 2.

**Table 2:** Outcomes of o-NA and p-NA reduction using magnetic nanocatalyst.

Nitroaromatics	Conversion (%)	R^2^	K_app_ (s^−1^)	Completion time (s)
O-nitroaniline	97.23	0.980	8.39 × 10^–2^	27
P-nitroaniline	97.04	0.969	5.48 × 10^–1^	8

In Table 3, the performance of the prepared nanocatalyst was compared to the other recent catalysts. From Table 3, obviously can be seen that the reaction time declined from 90 to 27 s for o-nitroaniline and from 40 to 8 s for p-nitroaniline that indicated that the CuFe_2_O_4_@CQD (citric acid) surpassed the CuFe_2_O_4_ nanoparticles. In addition, the apparent constant rate of the reduction reaction for o-nitroaniline and p-nitroaniline was increased from 3.48 × 10^–2^ to 8.39 × 10^–2^ and from 7.98 × 10^–2^ to 5.48 × 10^–1^, respectively.

**Table 3:** Comparison of the constant rate of other catalysts used for reduction of o-NA and p-NA.

Catalyst	Catalyst amount	K_app_ (s^−1^)	Time	Nitroarene	References
20%V dopped-Bi_2_(O, S)_3_	10 mg	34.4 × 10^–3^	150 s	o-Nitroaniline	^74^
Pd/CoFe_2_O_4_/chitosan	4 mg	–	65 s	o-Nitroaniline	^45^
CoMn_2_O_4_/APTPOSS@FPS	1 mg	1.83 × 10^–2^	100 s	o-Nitroaniline	^71^
CoMn_2_O_4_/APTPOSS@FPS	1 mg	1.83 × 10^–2^	100 s	o-nitroaniline	^71^
Au/SiO_2_-shell/Fe_3_O_4_-core	3 mg	4.1 × 10^–3^	4.2 min	o-Nitroaniline	^72^
SiO_2_@CuxO@TiO_2_	10 mg	0.018	150 s	o-Nitroaniline	^75^
Ag NPs	0.50 mL	2.43 × 10^–3^	540 s	o-Nitroaniline	^76^
CeO_2_	0.1 mg	–	60 s	o-Nitroaniline	^77^
GO/Au	5 mg	5.8 × 10^–3^	1320 s	o-Nitroaniline	^78^
Ag/kcc^−1^	10 mg	4.3 × 10^–3^	540 s	o-Nitroaniline	^79^
CuFe_2_O_4_	3.5 mg	3.48 × 10^–2^	90 s	o-Nitroaniline	^54^
CuFe_2_O_4_@CQD (gelatin)	3.5 mg	9.3 × 10^–2^	35 s	o-Nitroaniline	^80^
CuFe_2_O_4_@CQD (citric acid)	3.5 mg	8.39 × 10^–2^	27 s	o-Nitroaniline	This study
ZnO nanoparticles	10 mg/L	2.44 × 10^–2^	105 min	p-Nitroaniline	^81^
Ni/RGO	10 mg/50 mL	1.29 × 10^–2^	190 min	p-Nitroaniline	^82^
ZnO/CdO/RGO	1.2 mg/L	7.1 × 10^–3^	120 min	p-Nitroaniline	^83^
Ag nanoparticles	4 mg/L	8.52 × 10^–1^	13 min	p-Nitroaniline	^84^
CuFe_2_O_4_	3.5 mg	7.98 × 10^–2^	40 s	p-Nitroaniline	^54^
CuFe_2_O_4_@CQD (gelatin)	3.5 mg	2.89 × 10^–1^	13 s	p-Nitroaniline	^80^
CuFe_2_O_4_@CQD (citric acid)	3.5 mg	5.48 × 10^–1^	8 s	p-Nitroaniline	This study

To accurately evaluate the best-fabricated nanocatalyst to catalyze the ortho-NA and para-NA, the $K{^{\prime}}=K/m$ formula was used. Where $m$ is considered as the exact amount of used catalyst in the reduction in g or mg and $K$ as the calculated reaction constant rate. As we calculated, the constant rate of CuFe_2_O_4_@CQD as a nanocatalyst per unit was, $K{^{\prime}}=$ 8.39 × 10^–2^ (s^−1^)/3.5 (mg) and 5.48 × 10^–1^ (s^−1^)/3.5 (mg) for ortho-nitroaniline and para-nitroaniline, sequentially^71^.

### Analyze the recycle of nanocatalyst

**Figure 15:**
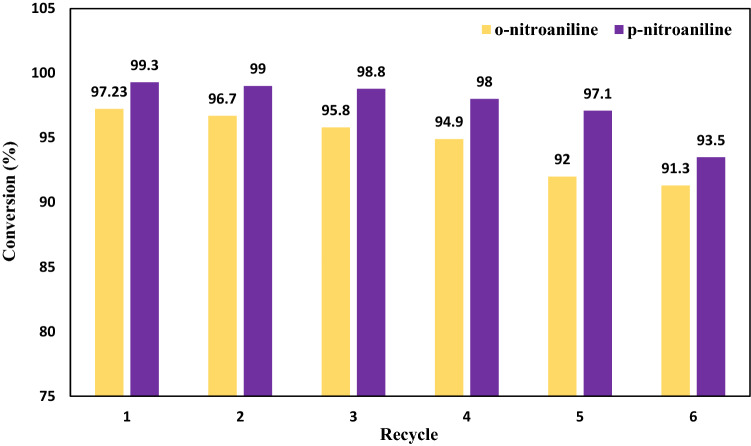
Recycle of nanocatalyst for up to 6 cycles.

The nanocatalyst’s performance was assessed for up to 6 cycles to further evaluate the catalyst’s reusability. Regarding this intention, in the first stage, the nanocatalyst was rinsed with ethanol and water after each reduction, and after reaction completion, it was dried utilizing a pump. As shown in Fig. 15, the fabricated catalyst functioned well based on the mentioned response time to reduce o-NA and p-NA. Therefore, during recycling, the catalyst conversion rate in the presence of nanocatalyst decreased from 97.2 to 91.3% for o-nitroaniline and 99.3–93.5 for p-nitroaniline, indicating high usability as well as stability of the prepared catalyst.

## Conclusion

As a result, a novel CuFe_2_O_4_@CQD nanocomposite as a heterogeneous nanocatalyst system was successfully fabricated and characterized. Characterization studies showed that CuFe_2_O_4_@CQD nanocomposite had cubic and spherical shapes, and their particle size was about 13.5 nm. The catalytic potential of CuFe_2_O_4_@CQD nanocatalyst was tested with NaBH_4_ towards reducing o-NA and p-NA. The catalytic activity of NPs was regenerated by adding CQDs of citric acid. In addition, the magnetic nanocatalyst converted o-NA to o-phenylenediamine and p-NA to p-phenylenediamine in just 27 and 8 s. Furthermore, it was found that the CuFe_2_O_4_@CQD nanocatalyst could be reused for up to six cycles.

In summary, the designed catalytic system showed valuable properties such as easy recovery, high stability, easy operation, long life, and excellent catalytic activity in a short reaction time compared to CuFe_2_O_4_ NPs. These outcomes conclude that CuFe_2_O_4_@CQD nanocatalyst had promising applications in catalysis, especially for wastewater treatment in the dyeing industry.

## Data Availability

All data generated or analysed during this study are included in this published article.
